# Altered fire regimes modify lizard communities in globally endangered Araucaria forests of the southern Andes

**DOI:** 10.1038/s41598-021-02169-3

**Published:** 2021-11-22

**Authors:** José Infante, Fernando J. Novoa, José Tomás Ibarra, Don J. Melnick, Kevin L. Griffin, Cristián Bonacic

**Affiliations:** 1 Fauna Australis Wildlife Laboratory, Department of Ecosystems and Environment, School of Agronomy and Forestry, Pontificia Universidad Católica de Chile, Avenida Vicuña Mackenna 4860, 7820436 Santiago, Chile; 2 Programa de Doctorado en Ecosistemas Forestales y Recursos Naturales & Instituto de Conservación, Biodiversidad y Territorio, Facultad de Ciencias Forestales y Recursos Naturales, Universidad Austral de Chile, Casilla 567, Valdivia, Chile; 3 ECOS (Ecosystem-Complexity-Society) Co-Laboratory, Center for Local Development (CEDEL) & Center for Intercultural and Indigenous Research (CIIR), Pontificia Universidad Católica de Chile, Villarrica Campus, O’Higgins 501, Villarrica, Chile; 4 Millennium Nucleus Center for the Socioeconomic Impact of Environmental Policies (CESIEP) & Center of Applied Ecology and Sustainability (CAPES), Pontificia Universidad Católica de Chile, Avenida Vicuña Mackenna 4860, 7820436 Santiago, Chile; 5 Department of Ecology, Evolution and Environmental Biology, Columbia University, New York, NY 10027 USA; 6 Department of Earth and Environmental Sciences, Columbia University, Palisades, NY 10964 USA

**Keywords:** Fire ecology, Climate change, Climate-change impacts, Herpetology, Ecological modelling, Conservation biology, Biodiversity

## Abstract

Wildfire regimes are being altered in ecosystems worldwide. The density of reptiles responds to fires and changes to habitat structure. Some of the most vulnerable ecosystems to human-increased fire frequency are old-growth *Araucaria araucana* forests of the southern Andes. We investigated the effects of wildfires on the density and richness of a lizard community in these ecosystems, considering fire frequency and elapsed time since last fire. During the 2018/2019 southern summer season, we conducted 71 distance sampling transects to detect lizards in Araucaria forests of Chile in four fire “treatments”: (1) unburned control, (2) long-term recovery, (3) short-term recovery, and (4) burned twice. We detected 713 lizards from 7 species. We found that the density and richness of lizards are impacted by wildfire frequency and time of recovery, mediated by the modification of habitat structure. The lizard community varied from a dominant arboreal species (*L. pictus*) in unburned and long-recovered stands, to a combination of ground-dwelling species (*L. lemniscatus* and *L. araucaniensis*) in areas affected by two fires. Araucaria forests provided key habitat features to forest reptiles after fires, but the persistence of these old-growth forests and associated biodiversity may be threatened given the increase in fire frequency.

## Introduction

Wildfire regimes (e.g. fire frequency, extent, time since last fire, ignition sources)^1^ are being altered in many ecosystems worldwide by anthropogenic causes, including climate change^2, 3^. Over the past decades, the frequency of fires has increased significantly^4, 5^. Fires are powerful environmental filters that can shape entire animal communities by directly killing populations and by altering habitat structure and composition^6^. The effects of fires on forest ecosystems are influenced by the intensity and severity of fires, but also by the number of fire events and the time elapsed between fires^7, 8^. The density of reptiles have been shown to respond to fires and their consequential changes to habitat structure^9–11^. However, whether they decline or increase in number depends on the species’ functional traits and natural history^9, 12–16^.

Southern landscapes in Chile have experienced unprecedented and devastating fires in recent years^17^. One of the most iconic and vulnerable forest ecosystems that has suffered an increase in forest fires in the southern Andes are old-growth *Araucaria araucana* forests (commonly named “pewen”, “araucaria” or “monkey puzzle tree”). Araucaria forests have been shaped by a mixed-severity and low frequency natural fire regime (one each 7 years on average)^18, 19^. Mixed-severity fires includes both low-severity surface fires and high-severity fires or stand-replacing events^19^. Ignition source are typically volcanic eruptions, and widespread high-severity events have been infrequent (62 years interval on average). There is also little evidence that indigenous people (Mapuche and Pewenche people), in the past, used to manage Araucaria forests with fire^20–22^. However, increases in anthropogenic fire ignitions associated with an extensive road network for intensive agriculture, exotic pine plantation forestry (*Pinus radiata*), livestock farming, droughts and tourism have dramatically altered the frequency and intensity of forest fires in southern Chile^19^. Climate data projections forecast increasing forest fire frequency and intensity due to climate change in the temperate rainforest of Chile^4, 23^. Forest fires are now considered as a concerning threat in this country, and their ecological consequences need to be studied^24^.

The species comprising reptile communities can shift in response to fire, given that some species may benefit from new habitat conditions, while others disappear or decrease in their density^14, 25^. Furthermore, species composition will also depend on the time elapsed since last fire, given the changes associated with the successional stages of vegetation^26–28^. Previous research has shown that reptiles can differ in their preferences on vegetation structure and complexity, from open grasslands to closed forests^29–32^. Lizards are some of the least studied vertebrates in their ecology and population status in Chile, and their response to forest fires is largely unknown^33^. Previous studies showed that at least four Chilean lizard species can persist in exotic pine plantations, and that the community changed according to the plantation age and habitat openness^30^, with ribboned lizards (*Liolaemus lemniscatus*) thriving in new plantation stands. On the contrary, an earlier study observed slender lizards (*Liolaemus tenuis*) being more abundant in native continuous forests and remnant fragments than inside pine plantations in southern Chile^34^. The occurrence and density of these lizards may depend on the stage of ecological succession after the disturbance and related structural changes in the vegetation^35^.

A key habitat component for biodiversity, and particularly for lizards, is coarse woody debris (hereafter CWD^36, 37^). CWD generates shelter, denning sites, foraging and thermoregulation substrate for lizards^36, 38^. CWD is also heavily impacted by forest fire frequency and intensity, as fires both create and consume CWD, which acts as woody fuel^39^. An assessment of the effects of forest fires on a lizard community should include this habitat component, which is modified by fires and can simultaneously act as an important resource for several coexisting species.

In this study, we investigated the effects of recent wildfires on the density and richness of a lizard community in the threatened Araucaria forests of southern Chile, considering fire frequency and elapsed time since last fire. We also investigated habitat attributes, in terms of vegetation and CWD, associated with observed lizard density patterns in burned and unburned forests. We studied lizard species because (1) of the large proportion of endemic species in Chile^40^; (2) ecological knowledge regarding these species is limited in Chile^30, 34^; (3) many have poor conservation status or are under-assessed^41, 42^; (4) they occur in different habitat and microhabitat conditions^38^; and (5) they have been observed to be very sensitive to habitat modification^43, 44^. We expected that fires would modify lizard richness and density in Araucaria forests, determined by functional traits associated with natural history (e.g., arboreality, ground-dwelling). Particularly, we expected: (1) a negative impact on arboreal and CWD-dependent species in areas affected by more than one fire; (2) higher density of arboreal reptiles with longer time elapsed since last fire; and (3) a negative effect on overall lizard density and richness in areas affected by more than one fire. Information on the response of animal communities to altered wildfires in old-growth forests with low fire frequency is scarce, particularly in the global south. This study aims contributing to our understanding and predictions on global consequences of the widespread alterations of fire regimes.

## Results

**Figure 1:**
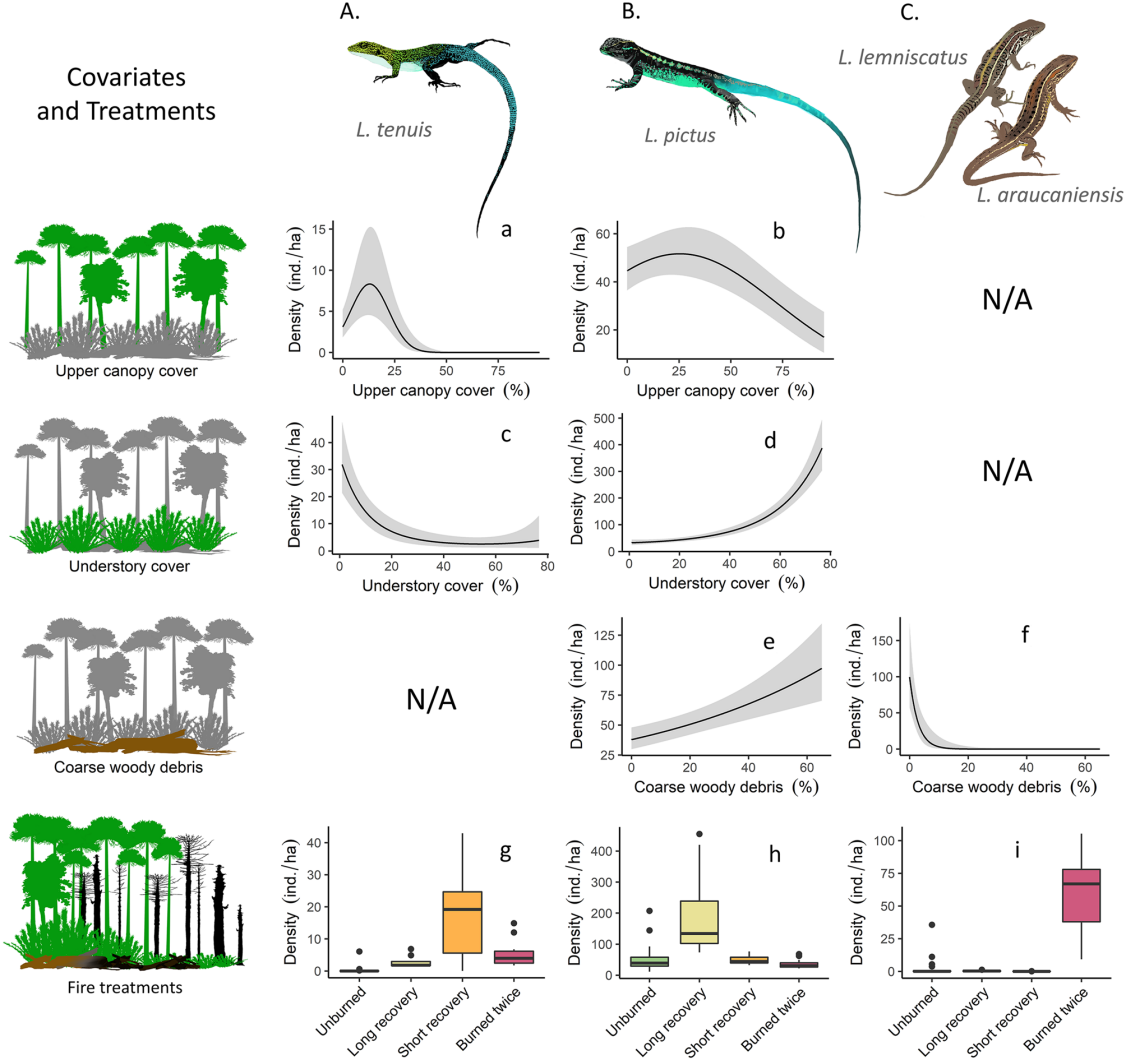
Modeling results for lizard density in relation to meaningful habitat covariates and “treatments'' for each lizard species. Leftmost column shows graphic representations of covariates and fire treatments (from top to bottom: upper canopy cover, understory cover, coarse woody debris cover, and fire treatments). Graphics are ordered by columns according to each modeled species: (**A**) slender lizard (*L. tenuis*); (**B**) painted lizard (*L. pictus*); and (**C**) araucana and ribboned lizards (“ground lizards”, *L. araucaniensis* and *L. lemniscatus*, respectively). Grey area shows estimated 95% confidence intervals. N/A (not applicable) indicate uninformative covariates according to AIC results for each species’ models.

We detected 713 lizards over 17,405 m of transects. During our surveys we recorded 7 lizard species: *L. tenuis* (99 detections), *L. pictus* (551), *L. lemniscatus* (8), *L. araucaniensis* (18), *L. chiliensis* (9, not modeled), *L. cyanogaster* (3 outside transects, not modeled) and *L. villaricencis* (1 outside transect, not modeled). We could not determine the species corresponding to 16 detections as the individuals fled before identification. We also detected 12 individuals that could not be differentiated between *L. lemniscatus* and *L. araucaniensis* due to their morphological similarities, sympatry and elusiveness. We merged the records of the latter species (hereafter, ground lizards) as both were observed to have similar habitat/microhabitat preferences, and to improve model convergence. All modeled species were previously detected in all the surveyed areas except for *L. araucaniensis*, which is documented for the first time in Tolhuaca NP in this study (see Fig. 1a–c for modeled species). Microhabitats varied among species, with *L. tenuis* and *L. pictus* more frequently observed using tree-derived substrates, particularly woody debris, while *L. lemniscatus, L. araucaniensis* and *L. chiliensis* were observed more frequently on the ground (Fig. S1).

### Species detectability and density

The detection of lizards decreased with distance, but distance distributions were different for each species (Fig. S2). Detection also varied with different combinations of covariates for each species. For *L. tenuis,* the sigma (σ) parameter varied negatively with temperature (β = − 0.142, SE = 0.078), as well as for *L. pictus,* although to a lesser extent (β = − 0.031, SE = 0.039). The sigma parameter of detection for *L. pictus* was also negatively associated with wind velocity during the transects (β = − 0.124, SE = 0.046), and positively associated with time (β = 0.020, SE = 0.03). Furthermore, wind velocity (β = 0.238, SE = 0.142) and time (β = − 0.204, SE = 0.175) were also selected for ground lizards’ best detection model. The estimates of species density also differed, with *L. pictus* being the most common (average = 52.2 ind./ha, SE = 4.98), followed by *L. tenuis* (average = 2.41 ind./ha, SE = 0.753), and lastly ground lizards, with an average density below 1 individual per hectare in the whole study area. Density estimations varied greatly with covariates. The quadratic form of canopy cover was a meaningful covariate for *L. tenuis* (highcan β = − 3.45, SE = 0.875; highcan^2^ β = − 4.97, SE = 1.72). This means that no canopy is as negative for the species as a cover higher than 25% (Fig. 1a), when leaving the remaining variables constant at their average values. Similarly, understory cover was also meaningful in its quadratic form (under β = − 0.794, SE = 0.145; under^2^ β = 0.31, SE = 0.145), but showed an overall negative association with density for this species (Fig. 1c). *L. pictus* density was also found to vary with the percentage of upper canopy cover, but the quadratic form had a stronger effect than the linear form (highcan β = 0.03, SE = 0.08; highcan^2^ β = − 0.17, SE = 0.049), meaning that higher densities were observed with covers up to 50% (Fig. 1b). For this species, the understory cover was also meaningful in both its linear and quadratic forms and positively associated with density (under β = 0.53, SE = 0.07; under^2^ β = 0.13, SE = 0.04; Fig. 1d). Furthermore, *L. pictus’* best model showed coarse woody debris cover as a meaningful variable with a positive association (β = 0.26, SE = 0.06; Fig. 1e). Finally, the best models for ground lizards only showed coarse woody debris cover to be a meaningful covariate, which was negatively associated with density (β = − 6.21, SE = 1.74; Fig. 1f).

### Wildfire regime effect

**Figure 2:**
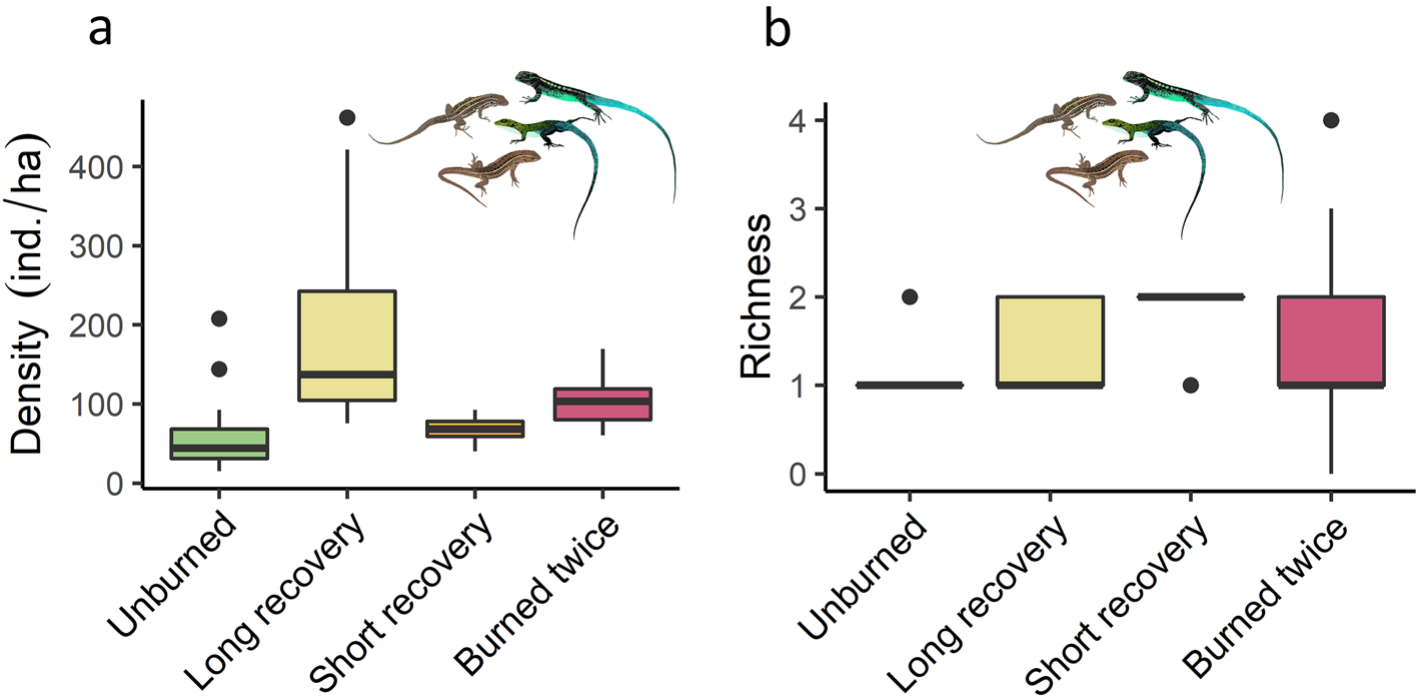
Total density (**a**) and richness (**b**) in each treatment for the lizard community in Araucaria forests from the Araucarias Biosphere Reserve (ABR) in the Andes of La Araucanía Region, Chile. Density estimates are based on predictions from best HDS models for each species. For richness results we used data from the 7 species detected.

The density of each lizard species varied according to number of fires (1 or 2) and the time of recovery in our “treatments”. *L. tenuis* was more abundant in the short-term recovery treatment (burned 2015), and a post-hoc test showed a marked difference of this treatment with the burned twice treatment (p = 0.0016, Fig. 1g). The most abundant species, *L. pictus*, displayed higher density in the long-term recovery treatment (burned 2002), being different from all other treatments (p < 0.0001, Fig. 1h). Inversely, ground lizards were most abundant in the burned twice treatment, which contrasts with their low abundance in the unburned forest (p = 0.043, Fig. 1i). For the lizard community, the total density of lizards showed a similar pattern to that of *L. pictus*, with higher densities in the long-term recovery treatment, and being different from all other treatments, according to Tukey’s range test (p < 0.0001, Fig. 2a). Finally, species richness was relatively low on average for all treatments, ranging from 0 to 4 species and averaging 1 to 2 per transect among treatments (Fig. 2b). According to Tukey’s range test, the short-term recovery treatment had the highest richness on average, showing marked differences with the unburned condition (p = 0.003).

## Discussion

Understanding the consequences of altered wildfire regimes on terrestrial biodiversity is critical, as these disturbances become more frequent at a global scale^45^. Here we reported the consequences of an altered fire regime on the understudied lizard community from the endangered *Araucaria araucana* forests in southern Chile. Our results suggest that both the density and richness of the studied lizard community are impacted by wildfire frequency and time of recovery, mediated by the modification of habitat structure. Importantly, the lizard community varied from a dominant arboreal species in unburned and long-recovered stands, to a combination of ground-dwelling species in areas affected by two successive fires.

### Lizard density after fire

Overall, fire disturbance favored the density of lizards, which increased with elapsed time since last fire. Previous studies show similar patterns of increasing density of reptiles with forest recovery^29, 46^; however, contrary to these, we observed relatively lower lizard density in unburned old-growth forests. The observed pattern could be related to an increase in thermoregulation opportunities after fires^16^, and unsuitable conditions for the modeled reptiles in long undisturbed forest stands. For example, although *L. pictus* was the most abundant species in unburned stands, its highest density was found in burned Araucaria forests that had experienced long recoveries. To compensate for poorer thermal conditions, high-altitude populations of *L. pictus* were described as spending more time thermoregulating^47^, increasing their need for thermally suitable microhabitats (e.g. CWD^48^). Fire events in the studied high-altitude Araucaria forests would generate thermoregulatory opportunities for lizards^49^, by creating CWD and a more open canopy, allowing greater light and solar radiation inside the forest and increasing the growth and reproduction of *L. pictus*. This process would also explain the higher density of the arboreal *L. tenuis* in burned forests with short recovery times, in relation to undisturbed forests. In our study, we detected both species in all burned treatments, however, their populations declined considerably in the burned twice condition, with *L. pictus* almost being extirpated.

On the other hand, ground lizards (*L. araucaniensis* and *L. lemniscatus*) displayed an opposite response, their highest density was found in the burned twice treatment, while going undetected in the unburned and burned once treatments. A previous study also observed higher densities of *L. lemniscatus* in more open forests^30^, explained by low levels of sunlight and heat reaching the surface, which would negatively affect ground-dwelling reptiles^50^. Hence, the simultaneous positive response to fire by arboreal and ground lizards resulted in the overall increase in density of lizards among fire treatments, but the composition of species varied considerably among them.

### Lizard richness after fire

Our data showed slight variations in species richness among treatments, which is consistent with previous reptile studies in temperate forests^46^. However, when observed at a larger scale (the entire study area), fire-diversity (i.e. patches of forest with different fire-ages) promoted lizard richness, given the differences in lizard communities among fire “treatments”. At a large scale, fire seems beneficial for increasing the richness of reptiles^51^. Furthermore, it has been suggested that landscapes with greater fire-diversity support greater reptile diversity (but see^52^). For example, in the Cerrado biome of Brazil, half of the species have been associated with unburned areas, while the other half have been associated with burned areas^16^. Here, we observed a clear differentiation in species composition between the burned twice treatment (where *L. araucaniensis* and *L. lemniscatus* dominated) and the long and short-term recovery treatments (where *L. pictus* and *L. tenuis* dominated).

### Habitat adaptations driving the response of lizards to fire

Our analysis showed that variation in habitat structure was sufficient to model most of the heterogeneity in lizard density in our study area (see best models in Table 1). This is consistent with other studies, where differences in reptile community structure among surveyed sites correlated with variation in vegetation structure; the latter being driven by fires^14^. Additionally, the response to habitat structure for each of our modeled species was different due to differences in their natural histories and functional traits^32^, which, in turn, segregated the species into distinct post-fire successional specialization categories^27, 35^.

**Table 1:** Statistics for best-ranked models (ΔAIC < 2) of lizard species in Araucaria forests of La Araucanía Region, Chile.

Species	Formula	Delta	AICwt	R^2^	nPars
*L. tenuis*	***d*(Temp) *D*(highcan + highcan**^**2**^** + under**^**2**^** + under)**	0.00	0.63	0.91	7
	*d*(Temp) *D*(CWD + highcan + highcan^2^ + under^2^ + under)	1.70	0.27	0.91	8
*L. pictus*	***d*(wind + time + temp) *D*(CWD + highcan + highcan**^**2**^** + under**^**2**^** + under)**	0.00	1	1.00	11
*L. araucaniensis*/*L. lemniscatus*	***d*(wind + time) *D*(CWD)**	0.00	0.27	0.71	5
	*d*(wind + time) *D*(CWD + highcan)	0.38	0.23	0.72	6
	*d*(wind + time) *D*(CWD + under)	1.41	0.14	0.72	6
	*d*(wind + time) *D*(CWD + highcan + under)	1.66	0.12	0.73	7

We show models formulas indicating detection covariates (*d*) and density covariates (*D*). Best model is highlighted in bold for each species.

For example, *L. tenuis* was likely less abundant in forests with longer recovery times (i.e. higher canopy and understory cover) than *L. pictus* due to the tolerance of the latter towards colder environments. *L. pictus* is more adapted to structurally complex and cold temperate forests of south-central Chile, occurring further south than *L. tenuis*^53^. Our models predicted that *L. pictus* can be more abundant with higher levels of upper canopy cover (i.e. more shade) and higher understory cover than *L. tenuis* (Fig. 1a–d), which could be explained by their differentiation in thermal niches^54^.

Furthermore, we believe that the negative association found by our models between ground lizards’ density and CWD cover was given by the specialization of these species to open habitats with low structural complexity, rather than by a detrimental effect of CWD. The negative correlation would have arisen due to the decrease of CWD cover caused by a higher fire frequency in that treatment^39^. In fact, some ground lizard individuals were observed exploiting remaining CWD, using it as shelter and for basking. Ground lizards would have increased their density in the burned twice treatment due to the transformation of the forest into a grassland/shrubland habitat following the increase in fire frequency^55^, which likely favored these reptiles because of their high thermal requirements^56^.

Additionally, habitat response to fire can vary spatially, resulting in geographical variations in lizard responses to these disturbances^26, 57^. Therefore, the effects of altered fire regimes on our modeled species should also be studied in other ecosystems and regions. Finally, the response of the lizard community to fire frequency and elapsed time since last fire will be subject to the historical fire regime in the landscape, that shaped their evolutionary trajectories and adaptations. Hence, the resulting community structure would depend on species’ differences in natural history, diet, thermal ecology, among others^16^.

## Limitations and future directions

Although our results suggest that increasing the frequency of fires may not be detrimental (or it may even be beneficial) to some lizards in terms of richness and density, we consider that these results may represent only the most fire-resilient reptiles in Araucaria forests. For example, none of the lizards detected are considered old-growth forest specialists. Old-growth forest species are known to be very sensitive to fire events and changes in fire regimes^58^. We do not know the response of rarer and more elusive lizards that could be considered old-growth forest (or undisturbed forest) specialists, such as *P. torquatus,* to our fire treatments^38^. For this and other elusive species, future research may test alternative methodologies (i.e. funnel traps, drift fences, pitfall traps and camera traps), that could increase their detection.

Moreover, the positive response to fire of most of the studied lizards in our study area may be jeopardized by the trend toward higher fire frequency and an increase in megafires^51, 59^. It is likely that more fires will further reduce population numbers, mainly from arboreal and CWD-dependent species, and probably extirpate them. Forest habitat loss and modification have caused the extinction of native herpetofauna from a wide extension of south-central Chile. *L. pictus* has not been detected in the intermediate depression of Chile since 1934^60^, which is likely due to forest habitat loss^53^.

A manipulative experiment, such as those from prescribed fires^8^, may allow to control possible confounding variables rising from geographic differences and increasing sampling units and treatments. However, prescribed fires are illegal in native forests of Chile, and due to the relatively novelty of fire regime changes in temperate forests of the southern Andes, it is not yet possible to survey Araucaria forests burned three or more consecutive times, nor forests with very diverse fire-ages. Hence, landscape-scale natural experiments are the only current option to investigate the consequences of fires on this forest ecosystem. Indeed, we sampled all available area for the long-recovered treatment. Future research should focus on surveying lizards in our treatments in a multi-season study to better understand the dynamics of reptile assemblages after fires and, if possible, to survey reptiles in Araucaria stands burned more than twice.

## Implications and conclusions

This is the first study showing how wildfires modify the lizard community of Araucaria forests in southern Chile, considering both fire frequency and time elapsed since last fire. The structure of the resulting community depended on habitat structure and species’ functional traits. Our results provide evidence of the resilience of the detected species after fire disturbances. Two fires could even be beneficial to the threatened lizard *L. araucaniensis.* However, informed inferences about conservation outcomes should consider different taxa, as they may respond differently to these disturbances.

The thick bark of Araucarias allows them to resist severe fire damage^61^, while the co-occurring *Nothofagus* species are very sensitive to fire^22^. The persistence of Araucarias after fires is important for the resilience of animal communities that depend on tree-derived substrates, in particular, CWD, which has been found to be essential to the recovery of forest reptile populations^37^. Araucaria forests granted key habitat features to forest reptiles after fire disturbances, but the persistence of these old-growth forests and associated biodiversity may be threatened by the current upward trend in fire frequency.

## Materials and methods

### Study area

**Figure 3:**
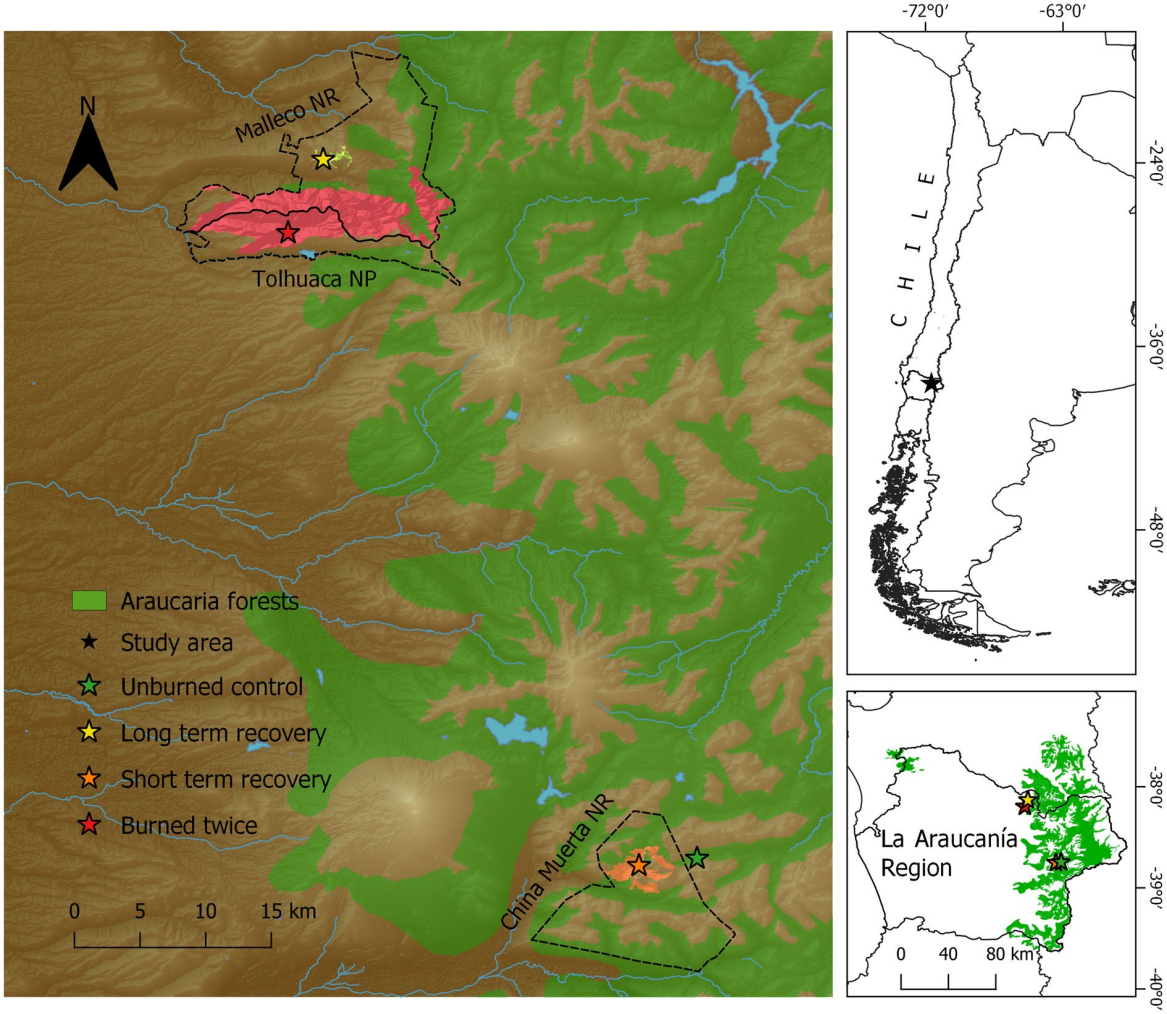
Study area map, located in the Araucarias Biosphere Reserve (ABR) in the Andes of La Araucanía Region, Chile. Fire “treatments” are shown in red, orange, yellow and green colors. We also show the approximate extent of araucaria forests affected by fires around our sampling areas. Green area shows *Araucaria Araucana* distribution in Chile^66^. Brown shading represents elevation, with higher elevations in lighter brown, and ranging from approximately 400 to 3000 m.a.s.l. All treatments were located between 1014 and 1469 m.a.s.l. Protected areas are delimited by dashed black lines.

The study was conducted in an area adjacent to the China Muerta National Reserve (11,170 ha), inside the Malleco National Reserve (16,625 ha), and inside the Tolhuaca National Park (6408 ha), all of which belong to the Araucarias Biosphere Reserve (ABR) in the Andes of La Araucanía Region, Chile (38°–39°′S 71°W; UNESCO 2010; Fig. 3). Elevation in the ABR ranges from 200 to 3747 m.a.s.l. with forests up to ~ 1500 m.a.s.l. The Araucaria forests are found at over 1000 m.a.s.l.^62^ and are dominated by millennia-old *Araucaria araucana* and species of the *Nothofagus* genus^63, 64^, with *Chusquea* spp. dominating the understory^65^. The study area possesses a temperate climate with a short dry season (January–March) and average annual rainfall of 1945 mm^66^.

### Fire treatments

**Figure 4:**
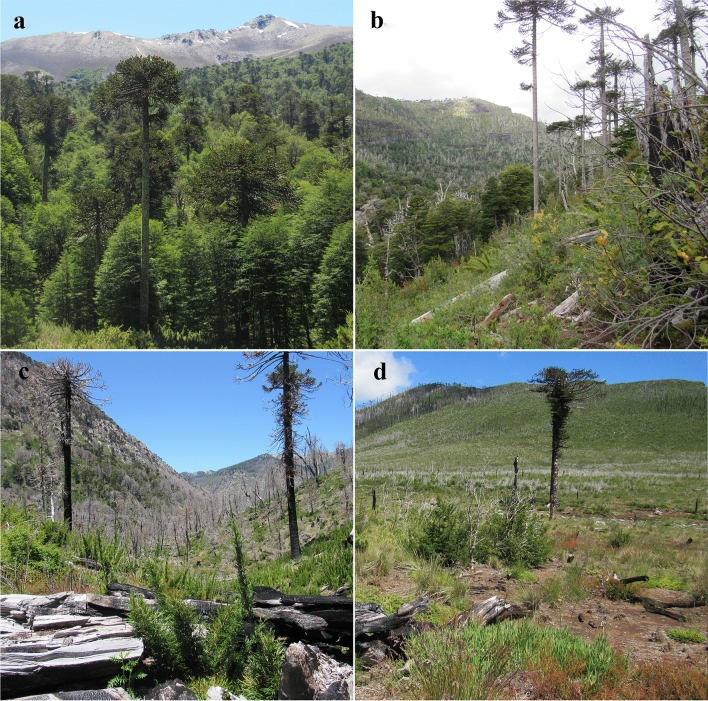
Forest conditions of the four “treatments”: (**a**) unburned control [old growth forest with 5,000 year old araucarias]; (**b**) “long-term recovery” (16 years) [abundant CWD, evident understory and canopy regrowth]; (**c**) “short-term recovery” (3 years) [abundant CWD, understory and canopy regrowth is still scarce]; (**d**) “burned twice” (3 years of recovery) [scarce CWD, understory regrowth is scarce and canopy almost nonexistent].

We selected four “treatments” according to their recent fire histories: (1) unburned control; (2) burned once with 16 years of recovery (hereafter, “long-term recovery”; close to control condition because it was burned once and experienced a relatively long recovery; burned in 2002); (3) burned once with 3 years of recovery (hereafter, “short-term recovery”; moderately disturbed because it was burned once and experienced a relatively short recovery; burned in 2015); and (4) burned twice with 3 years of recovery (hereafter, “burned twice”; highly disturbed due to the two successive fires and experienced a relatively short recovery; burned in 2002 and 2015; Fig. 4). Treatments were selected by satellite imagery of pre- and post-fire conditions and by field visits. Random assignment of treatments is impossible in our study system due to the illegality of igniting fire in native forests in Chile. These four treatment areas were located between 1014 and 1469 m.a.s.l. and separated by at least 3 km from the next closest treatment. Most of the recent fire events that occurred in our treatments are believed to be caused by human actions. Estimated severity of last fires in all three burned conditions was high, according to the assessment by the National Forestry Service^67^. Surveyed forests suffered crown fires with > 90% of trees and understory vegetation charred. A dense ash layer of ~ 30 cm deep covered the soil, and large holes in the ground caused by carbonized tree stumps occurred frequently. Moreover, root damage and total shrub vegetation loss occurred during the fires^67, 68^.

### Lizard community

The lizards reported to occur inside and adjacent to our study area comprise 14 species (5 endemic) in three genera, with 9 species not evaluated or data-deficient according to the IUCN and 5 species in a threatened category according to Chile’s Ministry of the Environment: the Painted lizard (*Liolaemus pictus*), Northern Painted lizard (*Liolaemus septentrionalis*), Slender lizard (*Liolaemus tenuis*), Whipping lizard (*Liolaemus chiliensis*), Ribboned lizard (*Liolaemus lemniscatus*), Araucana lizard (*Liolaemus araucaniensis*), Blue-Bellied lizard (*Liolaemus cyanogaster*), Volcán Villarrica lizard (*Liolaemus villarricensis*), Blue-paunched lizard (*Liolaemus coeruleus*), Elongate tree iguana (*Liolaemus elongatus*), Schroeder’s lizard (*Liolaemus schroederi*), Janequeo’s lizard (*Liolaemus janequeoae*), the Southern growler (*Pristidactylus torquatus*), and the Six-banded Big-headed lizard (*Diplolaemus sexcinctus*)^38, 69^. The species *L. septentrionalis* and *L. pictus* were recently suggested as being classified as distinct species^70^, but due to their phylogenetic proximity, unclear distributional delimitations/co-occurrence, and ecological and morphological similarities, we will consider them the same for the analyses (hereafter, *L. pictus* or painted lizard).

### Habitat sampling

At each of our four “treatments”, we established a ~ 3 km long transect with 40 vegetation plots separated by at least 125 m (11.2 m radius; 0.04 ha; Total n = 160 plots)^71^, which overlapped with reptile sampling units. Each plot was divided in four quadrants with the aid of two tape measures and we visually estimated: understory cover (%, vegetation height of 0.5–3 m), coarse woody debris cover (%, woody debris with diameter ≥ 7.5 cm), intermediate canopy cover (%, vegetation height of 3–5 m), and upper canopy cover (%, vegetation height > 5 m).

### Reptile survey

We conducted 71 transects in Araucaria forests in the La Araucanía Region of Chile and visually counted and measured the distance to all sighted lizards. The study was conducted in accordance with the protocols of the Scientific Ethics Committee of the Pontificia Universidad Católica de Chile and all field methods were carried out in accordance with national regulations. Distances were obtained using the Bosch GLM-20 laser measure. Surveys took place during December and January of the 2018/2019 summer season. Transects were conducted between 11:00 a.m. and 6:00 p.m. due to greater lizard activity, and when air temperature in the shade was higher than 15 °C^40^. Transect lengths averaged 245 m and ranged from 83 to 440 m, varying according to field conditions (i.e. terrain roughness, cliffs, etc.). Transect width was truncated following advice on distance sampling methods^72^, which varied by species. Minimum distance between transects was based on average lizards’ home-ranges^40^, to avoid recording same individuals in different sampling units.

### Data analyses

We standardized continuous variables to z-scores, to facilitate interpretation of the covariate coefficients and to improve model convergence. We tested the 3 density predictors for collinearity and, when variables were correlated (Pearson’s/Spearman’s│r│ > 0.7), we retained the predictor that conferred greater ecological meaning and ease of interpretation^73^.

### Species detectability and density

We analyzed transect data using a hierarchical distance sampling (hereafter, HDS) framework^74^, which allowed us to correct estimated lizard densities by their probability of detection based on distance and environmental covariates^75^. To estimate detection and density of lizards we used the HDS model described by Royle et al.^74^, which assumes the measurement of distance in discrete intervals, and that sample unit *i* has local latent abundance *Ni*. The model is formulated as follows:

$$N_{i} \sim Poisson(\uplambda_{i} )\;{\text{ for}}\;\;i = \, 1, \, 2, \, \ldots ,M$$

where λ_*i*_ is the abundance at site *i.* This model also assumes a multinomial distribution for the detection frequencies in each of the *J* distance classes, which is conditional to the latent abundance *N*_*i.*_

$$y_{ij } \sim {\text{ Multinomial}}\;(N_{i} ,\uppi_{ij} )\;{\text{for}}\;i = 1,2, \ldots ,M,\;{\text{and}}\;j = \, 1, \, 2, \, \ldots ,J$$

where *y*_*ij*_ is the observed count of individuals in distance class *j*, and π_*ij*_ is the multinomial cell probability for transect *i* in distance class *j*. These are computed by the integration of a detection function (e.g. “half-normal”, negative exponential, and hazard rate^72^) with scale parameter σ. Transect-specific covariates are used to model λ and σ parameters using the log link function.

We applied maximum-likelihood method through the ‘distsamp’ function from the ‘unmarked’ package^76^ for R software environment v3.6.1^77^. We first used Akaike’s Information Criterion (AIC) to identify the most adequate detection function for each lizard species^75^. To estimate the probability of detection of each species, we used a set of three covariates: time, wind velocity (m/s), and temperature (°C) at each transect. The last two variables were measured with a portable meteorological unit (Kestrel 4200, Kestrel-meters, Birmingham, MI). Time was considered because some of our species were observed to have unimodal or bimodal activity during the day^38^. We used a stepwise covariate selection procedure and then, using AIC, selected the top-ranked model to be included in the modeling of density for each species^73^. To model the density of each species we tested upper canopy cover, mid canopy cover, understory cover, and coarse woody debris cover, and added quadratic terms for vegetation cover variables to account for non monotonic relationships (i.e. higher or lower density in intermediate levels of vegetation cover^78^). We built a candidate set of models to obtain the best density models for each species based on model weights (wi) using AIC^79^. Models with ∆AIC ≤ 2 were considered to have similar support and were averaged to predict species density for each transect^71^.

### Wildfire regime effect

We evaluated the effects of recent wildfire regime in our study area as “burning treatments” on lizard species density and richness using generalized linear models (GLM) with Poisson error distribution. We used the predicted lizard densities (individuals per hectare) for each transect generated by the previous HDS models and analyzed the effect of the treatments for each species and for the total lizard density, while for richness we used the number of species detected in each transect. For these analyses, the ‘nlme’^80^ and ‘AICcmodavg’^81^ statistical packages were used in R^77^. Finally, we used Tukey’s range test through the function ‘glht’ (R package ‘multcomp’^82^) for post hoc testing in order to determine the differences between treatments.

### Ethical approval

Support and permissions to investigate in public protected areas (Tolhuaca National Park, Malleco National Reserve and China Muerta National Reserve) were given by National Forest Service of Chile (CONAF), and in private areas were given by the Pewenche Quinquén Community.

## Supplementary Information

Supplementary Information.

## Data Availability

The datasets generated during and/or analysed during the current study are available from the corresponding author on reasonable request.
