# Supplementary material for: Altered fire regimes modify lizard communities in globally endangered Araucaria forests of the southern Andes
Source: Sci Rep. 2021 Nov 22;11:22709. doi: 10.1038/s41598-021-02169-3 (PMC8609011; doi:10.1038/s41598-021-02169-3)
Supplement: Supplementary file 1 — Supplementary Information. [file 41598_2021_2169_MOESM1_ESM.pdf]

Consequences of altered fire regimes on lizard communities in globally endangered Araucaria forests of the southern Andes

José Infante*, Fernando J. Novoa, José Tomás Ibarra, Don J. Melnick, Kevin L. Griffin, Cristián Bonacic

*Corresponding author: joinfant@uc.cl

SUPPLEMENTARY INFORMATION

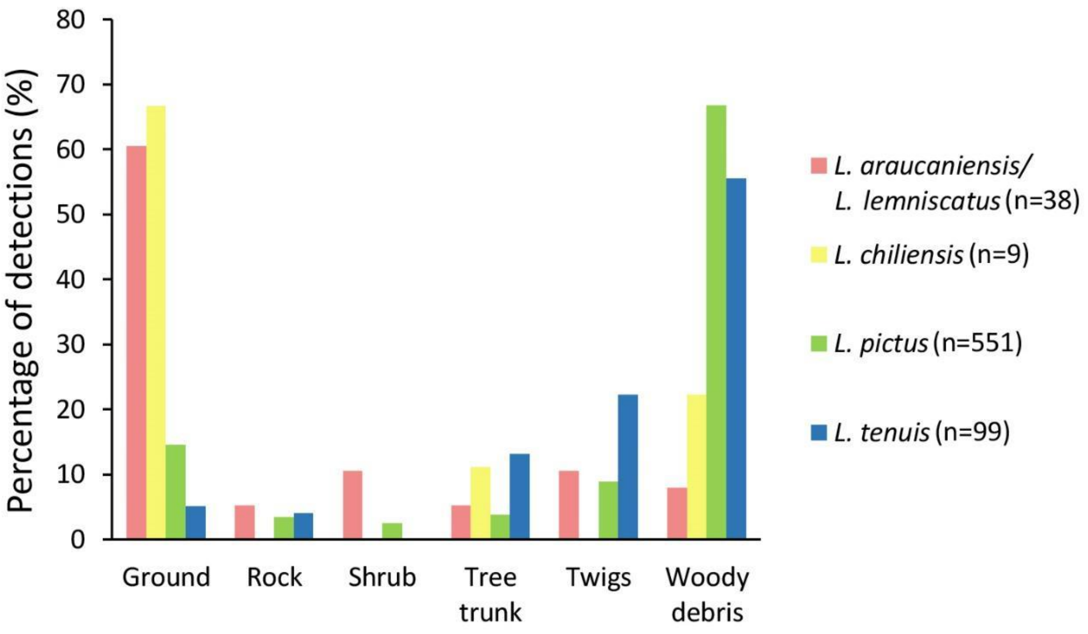

Figure S1. Percentage of lizard detections in microhabitats: ground, rock, shrub, tree trunk, twigs, and woody debris. Percentages are derived from total counts of each species.

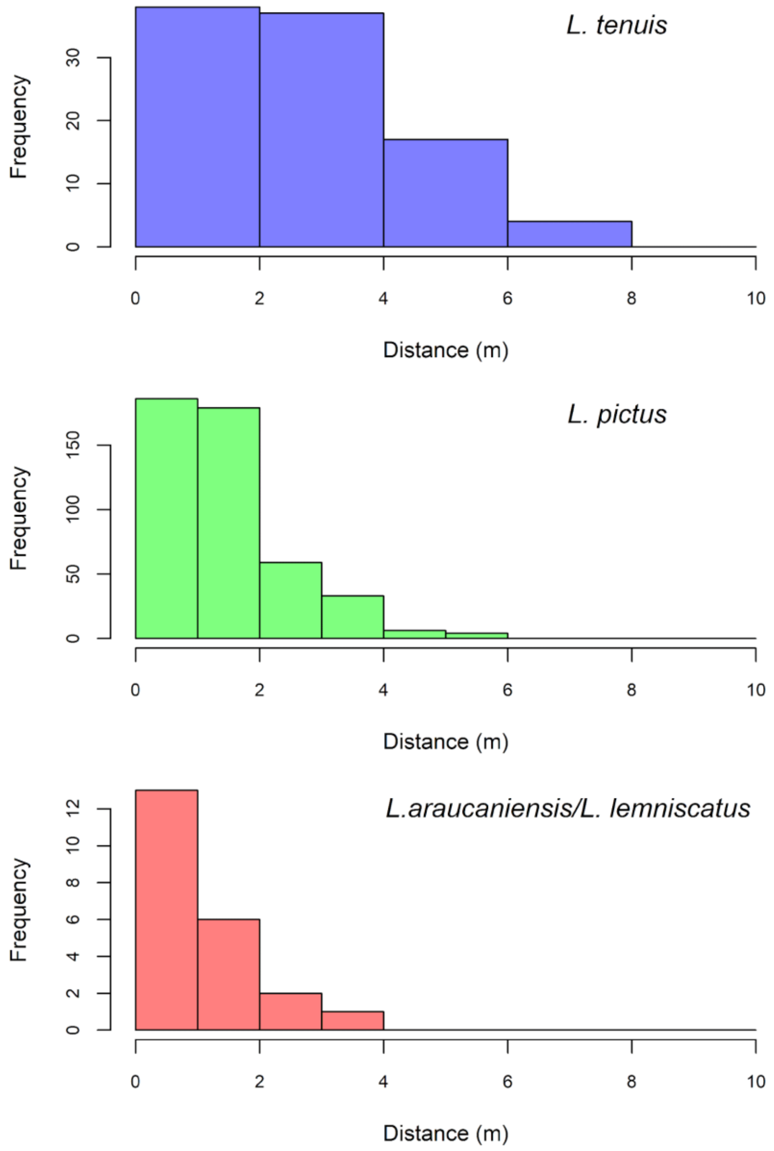

Figure S2. Histograms of lizard detections in relation to distance bands for *L. pictus*, *L. tenuis* and merged detections of *L. araucaniensis* and *L. lemniscatus*. According to our AIC analysis, the "half-normal" detection function was selected for *L. tenuis*, while hazard rate and negative exponential were selected for *L. pictus* and ground lizards, respectively.

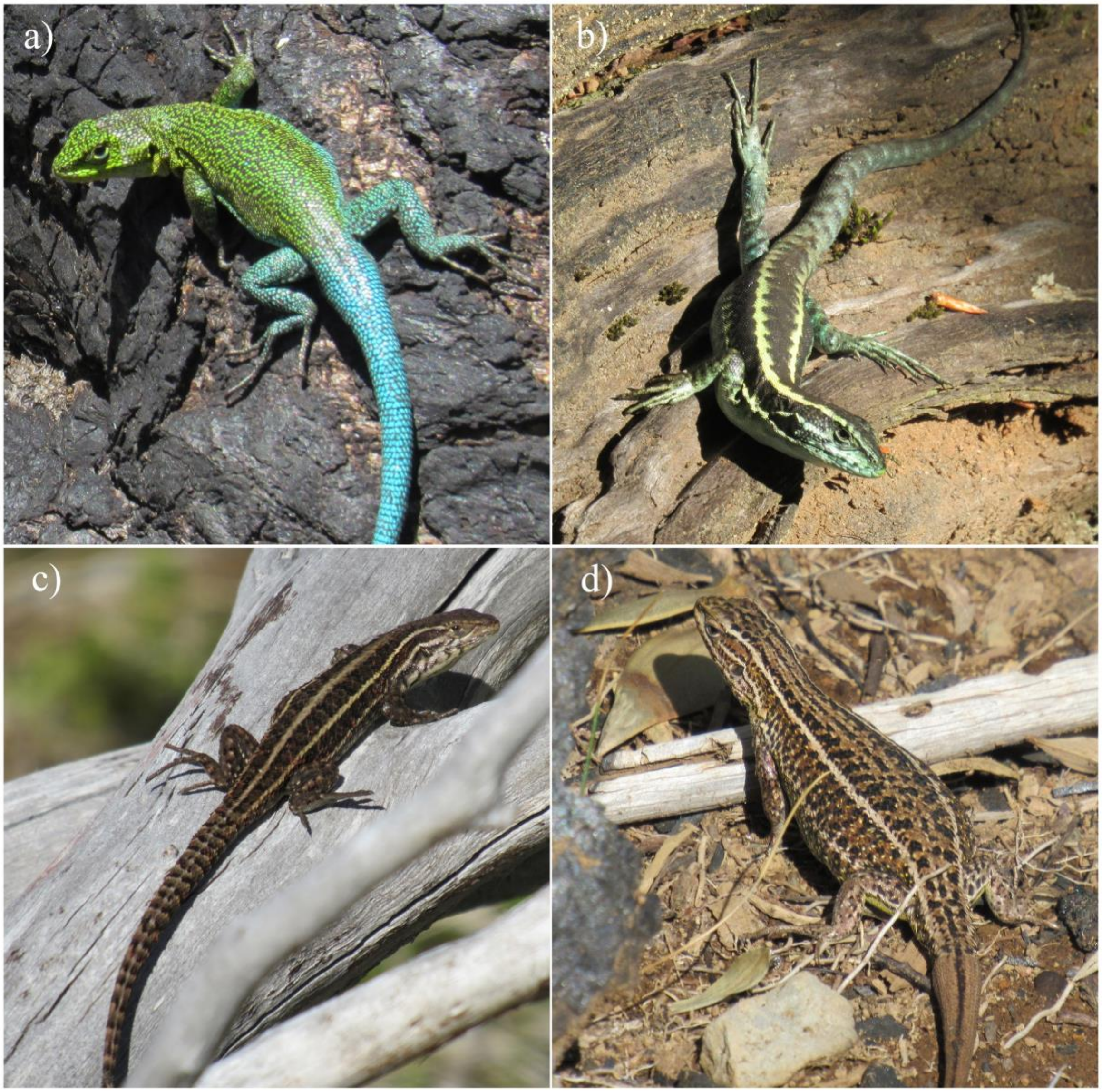

Figure S3. Modeled lizard species: a) *L. tenuis* (male) [slender shape; rounded dorsal scales; males half yellow-greenish colors and half blue-cyan colors], b) *L. pictus* [rounded and small dorsal scales; dorsum black with variable accompanying coloration: olive-brown, orange, red, green, and/or blue, with light colored stripes from eyes to base of tail and black triangular dots in zig-zag], c) *L. lemniscatus* [small size; slender shape; short limbs; triangular mucronate scales; dorsum brown with light yellow stripes from eyes to base of tail, a brown band from nape base to tail, and in males few light blue scales], d) *L.*

araucaniensis (gravid female) [small size; triangular mucronate scales; dorsum brown with light yellow stripes from eyes to base of tail, and a black vertebral line, usually segmented, from head to tail, accompanied by black triangular dots].

RESUMEN

Los regímenes de incendios están siendo alterados en distintos ecosistemas alrededor del mundo. Los reptiles han mostrado responder de forma significativa a los incendios y los cambios en la estructura de sus hábitats. Uno de los ecosistemas más vulnerables al incremento en la frecuencia de incendios inducidos por el ser humano son los bosques antiguos de *Araucaria araucana,* en los Andes del sur. Investigamos los efectos de incendios en la densidad y riqueza de la comunidad de lagartijas en estos ecosistemas, considerando tanto la frecuencia como el tiempo transcurrido luego del último incendio. Durante la temporada estival de 2018/2019, realizamos 71 transectos con muestreo de distancia de detección de reptiles en bosques de Araucaria en Chile dentro de cuatro "tratamientos" de incendios: 1. control no incendiado, 2. recuperación a largo plazo, 3. recuperación a corto plazo, y 4. quemado dos veces. Detectamos 713 lagartijas de 7 especies. Encontramos que la densidad y riqueza de lagartijas fue impactada por la frecuencia y tiempo de recuperación, mediado por la modificación de la estructura del hábitat. La comunidad de lagartijas cambió de una especie arbórea dominante (*L. pictus*) en ecosistemas no incendiados y de recuperación a largo plazo, a una combinación de especies terrestres (*L. lemniscatus* and *L. araucaniensis*) en áreas afectadas por dos incendios. Los bosques de Araucaria proveyeron características clave del hábitat para las lagartijas luego

de los incendios, pero la persistencia de los bosques antiguos y su biodiversidad asociada puede estar amenazada dado el incremento en la frecuencia de incendios.

Keywords

comunidad de reptiles, lagartijas, *Liolaemus*, *Araucaria araucana*, bosques de Araucaria, detrito leñoso grueso, ecología de incendios, crisis climática, modificación de hábitat.
